# UDP-glucuronosyltransferases 2A3 as a biomarker for ulcerative colitis and colon cancer

**DOI:** 10.3389/fgene.2024.1419755

**Published:** 2024-12-09

**Authors:** Hao Chen

**Affiliations:** Department of Surgical Oncology, The First Affiliated Hospital of Xi’an Jiaotong University, Xi’an, China

**Keywords:** *UGT2A3*, machining learning, ulcerative colitis, colon cancer, diagnostic

## Abstract

**Background:**

Ulcerative colitis has a serious impact on the quality of life of patients and is more likely to progress to colon cancer. Therefore, early diagnosis and timely intervention are of considerable importance.

**Methods:**

Gene expression data of active ulcerative colitis were downloaded from the Gene Expression Omnibus (GEO) database, and genes with significant differential expression were identified. Biochemical markers with diagnostic significance were selected through machine learning methods. The expression differences of the selected markers between colon adenocarcinoma (COAD) and healthy control groups in The Cancer Genome Atlas (TCGA) database were analyzed to evaluate their diagnostic value. In addition, the correlation between the selected markers and clinical indicators, as well as their predictive efficacy for the survival of COAD patients, was explored.

**Results:**

Through machine learning and LASSO regression analysis, *UGT2A3* was finally determined as a diagnostic marker for ulcerative colitis. It demonstrated high diagnostic accuracy in both the training set and the external validation set. Furthermore, *UGT2A3* was significantly downregulated in COAD tissues compared to normal control tissues. The ROC curve suggested that *UGT2A3* could serve as a diagnostic marker for COAD with excellent performance, achieving an AUC of 0.969. Immune infiltration analysis indicated a significant negative correlation between the expression of *UGT2A3* and neutrophils. Correlation analysis suggested a link between *UGT2A3* and the pathological classification of colon cancer. Survival analysis showed that *UGT2A3* is negatively correlated with OS, PPS, and RFS in colon cancer.

**Conclusion:**

The author identified *UGT2A3* as a diagnostic marker for ulcerative colitis through bioinformatics methods, and verified its significant downregulation in colon cancer, as well as its predictive role in the survival of COAD patients. These findings suggest that *UGT2A3* may serve not only as a diagnostic marker for ulcerative colitis and colon cancer but also as a potential prognostic indicator for colon cancer.

## 1 Introduction

Ulcerative colitis (UC) is an idiopathic chronic inflammatory bowel disease (Conrad et al., 2014). Patients with ulcerative colitis experience recurrent episodes of diarrhea, mucous, purulent bloody stools, and abdominal pain, which severely impact their quality of life (Van Assche et al., 2016; Ghosh et al., 2021), and increase the risk of colorectal cancer (Kvorjak et al., 2020). A population-based cohort study from Scandinavia showed that the probability of colorectal cancer (CRC) in UC patients was 1.66 times higher than that in the normal control group, and the risk of death in CRC patients with UC was 1.59 times higher than that in CRC patients without UC (Olen et al., 2020). The reason for the higher risk of colorectal cancer in UC patients is still unknown, and there are studies showing that RNA editing may mediate tumorigenesis in UC (Takahashi et al., 2023). Colorectal cancer associated with ulcerative colitis often represents a larger lesion area, more severe pathological staging, and a poorer prognosis compared to sporadic colorectal cancer (Zhang M. et al., 2023; Yashiro, 2014; Wang et al., 2017). Therefore, early diagnosis and timely intervention of UC play a crucial role in alleviating symptoms, enhancing quality of life, and slowing disease progression.

The diagnosis of ulcerative colitis mainly relies on clinical symptoms, endoscopic examination, and pathological biopsy, and some hematological indicators such as C-reactive protein (CRP), Erythrocyte sedimentation rate (ESR) can aid in UC diagnosis (Conrad et al., 2014; Ungaro et al., 2017), Certain fecal biochemical markers, such as calprotectin and S100A12, also exhibit diagnostic efficacy for UC (Liu et al., 2022; Mosli et al., 2015). Studies have developed diagnostic markers to improve UC diagnostic accuracy, with literature indicating high diagnostic accuracy for markers like CCL3, MMP3 and TIMP3 (Pan et al., 2023). However, a gap remains in identifying diagnostic markers that are effective for both UC and CRC. This study aims to screen biochemical markers of UC through bioinformatics methods, assess their expression and diagnostic efficacy in CRC, and provide insights for diagnosing both UC and CRC.

## 2 Material and methods

### 2.1 Data acquisition

The author obtained the Series Matrix File data files from the Gene Expression Omnibus (GEO) repository. The author separately obtained the expression matrices of health controls and active ulcerative colitis from GSE75214 and GSE48958, and merged the two expression matrices to obtain a combined dataset. Both datasets were derived from mucosal gene expression profiling of samples from active ulcerative colitis or healthy controls. The GSE75214 dataset includes 80 active UC mucosal samples and 11 healthy control mucosal samples, while the GSE48958 dataset includes seven active UC mucosal samples and eight healthy control mucosal samples. The two datasets used a unified annotation platform. A total of 87 active ulcerative colitis patients and 19 normal patients were included. The author downloaded the expression matrix of healthy control patients and active ulcerative colitis from GSE59071 and GSE16879 to serve as the external validation group. The GSE59071 dataset comprises mucosal gene expression profiles from 80 active UC patients and 11 healthy control samples. The GSE16879 dataset contains mucosal gene expression profiles from 41 active UC patients and six healthy controls. Gene expression and clinical data for colon adenocarcinoma (COAD) were also downloaded from The Cancer Genome Atlas (TCGA) database, comprising 453 tumor samples and 41 healthy controls. Additionally, the mucosal gene expression data for 101 colorectal cancer patients and 33 healthy control samples in the GSE83889 dataset were downloaded for further validation.

### 2.2 Identification of differentially expressed genes

The “limma” package was used to identify differentially expressed genes (DEGs) in the combined databases of GSE75214 and GSE48958. The “ggplot2” and “pheatmap” packages were applied to create volcano plots and heatmaps for visual representation of the DEGs.

### 2.3 Enrichment analysis

Clustering analysis provided by the “ClusterProfiler” package was employed to investigate the functional relationship of the DEGs. Functional analysis of genes was performed using GO and KEGG analysis methods. The p value and q value both below 0.05 were deemed to carry considerable importance.

### 2.4 Machining learning

Genes were identified through machine learning by applying the “glmnet” package for LASSO regression analysis. The λ tuning parameter was selected by 10-fold cross-validation using the function cv.glmnet from the same R package, while the α parameter, which balances the lasso and ridge penalties, was set to the default value 1. And SVM-RFE was implemented using the “e1071,” “kernlab,” and “caret” packages. Recursive Feature Elimination (RFE) was employed to identify the most relevant features for the predictive model. RFE is a technique that iteratively removes less significant features and constructs models using subsets of the remaining features to identify the best performing feature subset. The process was regulated by “rfeControl” using “caretFuncs,” specifying the use of cross-validation (cv) as the method to ensure robust model evaluation and reduce the risk of overfitting. The SVM with a radial basis function kernel (svmRadial) was used as the underlying prediction model. Genes identified through these algorithms were displayed in a Venn diagram using the “venn” package.

### 2.5 Evaluation of diagnostic value

The validation and diagnostic value of screened genes were assessed using the “limma” and “ggpubr” packages. Datasets GSE59071 and GSE16879 were used to validate expression differences between UC and healthy controls. The “pROC” package was utilized to construct ROC curves, calculate the area under the curve (AUC), and assess the diagnostic efficacy of the screened genes in the combined datasets (GSE75214 and GSE48958) and the external validation datasets (GSE59071 and GSE16879).

### 2.6 Immune infiltration

To determine the fraction of immune cells in tumors, the author applied a linear support vector regression-based method, CIBERSORT, to estimate the relative ratios of 22 different immune cell types. At the same time, CIBERSORT produces an empirical P-value for each sample and tests the null hypothesis that there are no cell types in the tested sample. Samples with a P-value ≥0.05 were eliminated in the following analysis. The author performed CIBERSORT in R and the source code of CIBERSORT (R version 1.03) was downloaded from the CIBERSORT website. Spearman correlation analysis was conducted to assess the association between gene expression and immune cell infiltration.

### 2.7 Validation in malignant tissues

The “limma” and “ggpubr” packages were used to evaluate gene expression differences between colorectal cancer and healthy controls, along with constructing ROC curves and calculating AUC values. Protein differential expression between colorectal cancer and normal controls was analyzed using data from the HPA database.

### 2.8 Correlation between genes and clinical and immune factors

The “limma” and “ggpubr” packages were employed to investigate correlations between screened genes and clinical indicators in colorectal cancer patients, including tumor staging, KRAS mutations, MSI status, and pathological classification. Spearman correlation analysis was conducted to explore associations between screened genes and immune checkpoints. To assess the prognostic relevance of screened genes in colon cancer patients, The author examined their survival value using the Kaplan-Meier database. All statistical analyses were performed in R (version 4.0), with p < 0.05 considered statistically significant.

## 3 Results

### 3.1 Differential gene identification and enrichment analysis

**FIGURE 1:**
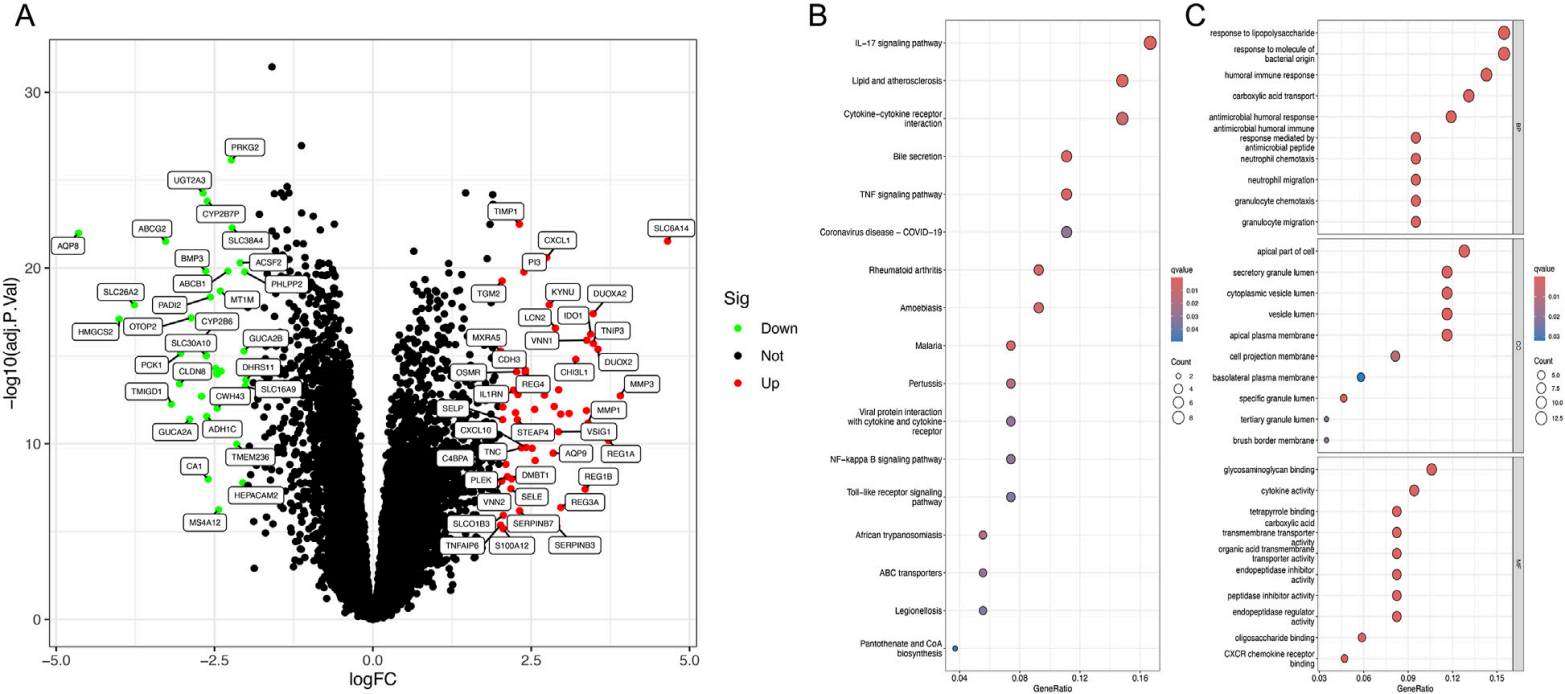
Identification of differentially expressed genes and enrichment analysis. **(A)** Volcano plot of DEGs between ulcerative colitis and normal tissues. **(B)** KEGG analysis of DEGs. **(C)** GO analysis of DEGs.

In the combined gene set, 52 upregulated and 35 downregulated genes were identified (Figure 1A). KEGG enrichment analysis suggested that these differential genes are primarily associated with the IL-17 signaling pathway, lipid and atherosclerosis, and cytokine-cytokine receptor interaction (Figure 1B). GO enrichment analysis indicated that the main functions of the differential genes are responses to lipopolysaccharide, response to bacterial-origin molecules, humoral immune response, carboxylic acid transport, antimicrobial humoral response, and apical cell components (Figure 1C).

### 3.2 Maching learning

**FIGURE 2:**
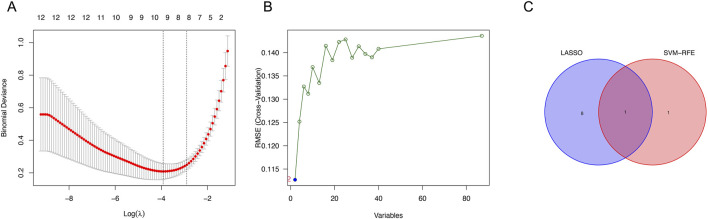
Identification of genes. **(A)** Expression coefficient maps of LASSO regression. **(B)** Cross-validation of the SVM model. **(C)** Venn diagram of LASSO and SVM-RFE screened genes.

From the LASSO analysis, nine genes were identified (Figure 2A), while the SVM-RFE algorithm identified two genes (Figure 2B). *UGT2A3* was determined from the intersection (Figure 2C).

### 3.3 Evaluation of diagnostic value

**FIGURE 3:**
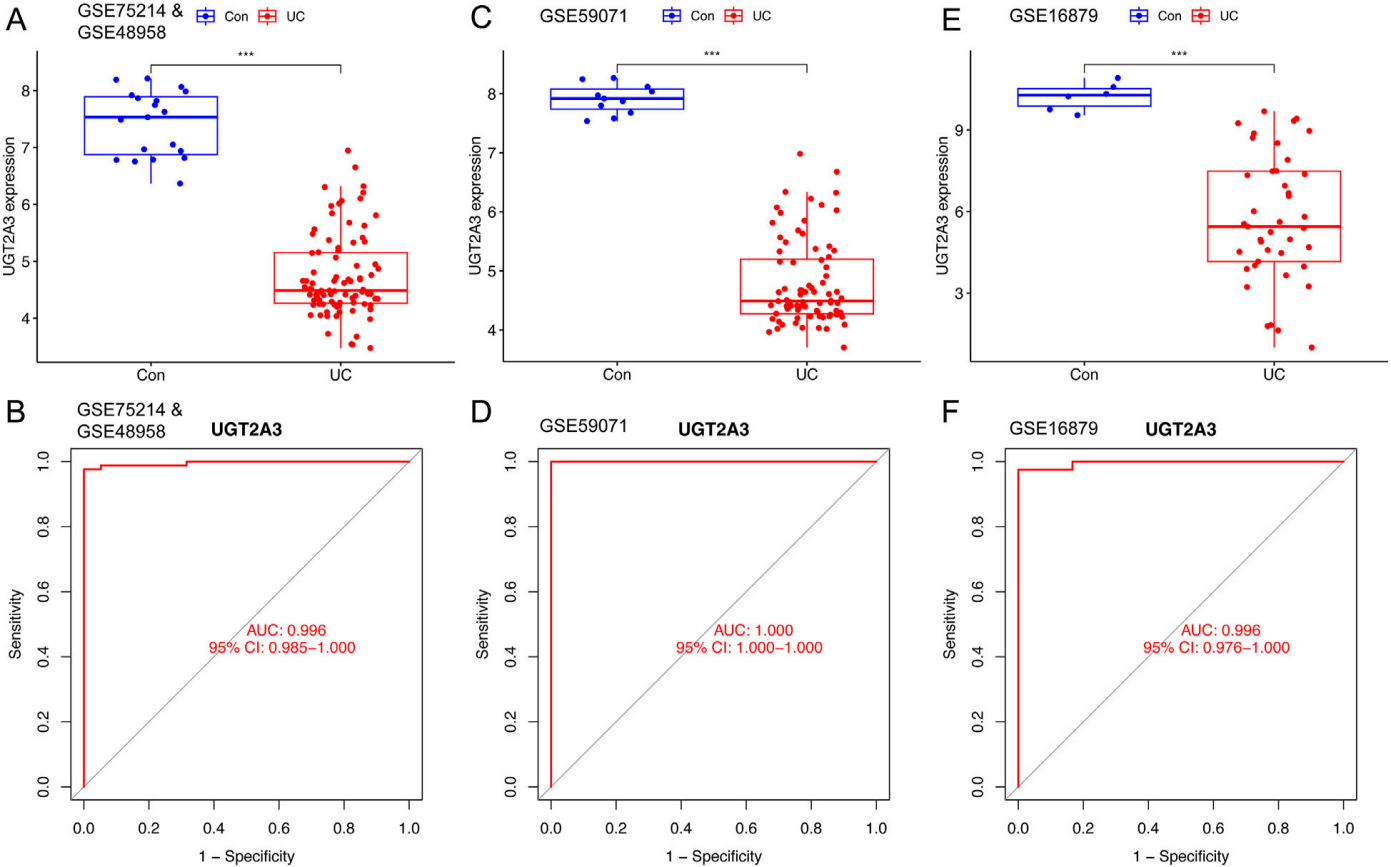
Expression differences and diagnostic value validation. **(A)** *UGT2A3* expression in ulcerative colitis and normal tissues of training set. **(B)** ROC curves of *UGT2A3* in training set. **(C)** *UGT2A3* expression in ulcerative colitis and normal tissues of GSE59071. **(D)** ROC curves of *UGT2A3* in GSE590971. **(E)** *UGT2A3* expression in ulcerative colitis and normal tissues of GSE16879. **(F)** ROC curves of *UGT2A3* in GSE16879. (*p < 0.05; **p < 0.01; ***p < 0.001).

The author assessed the diagnostic value of *UGT2A3* in the combined experimental dataset and in GSE59071 and GSE16879. In the combined dataset, *UGT2A3* showed significant expression differences, with an area under the ROC curve (AUC) of 0.996 (95% CI: 0.985–1.000). In the validation datasets, *UGT2A3* exhibited significant expression differences between active ulcerative colitis and healthy controls (Figures 3C, E), with AUCs of 1.000 (95% CI: 1.000–1.000) and 0.996 (95% CI: 0.976–1.000), respectively (Figures 3D, F).

### 3.4 Immune infiltration

**FIGURE 4:**
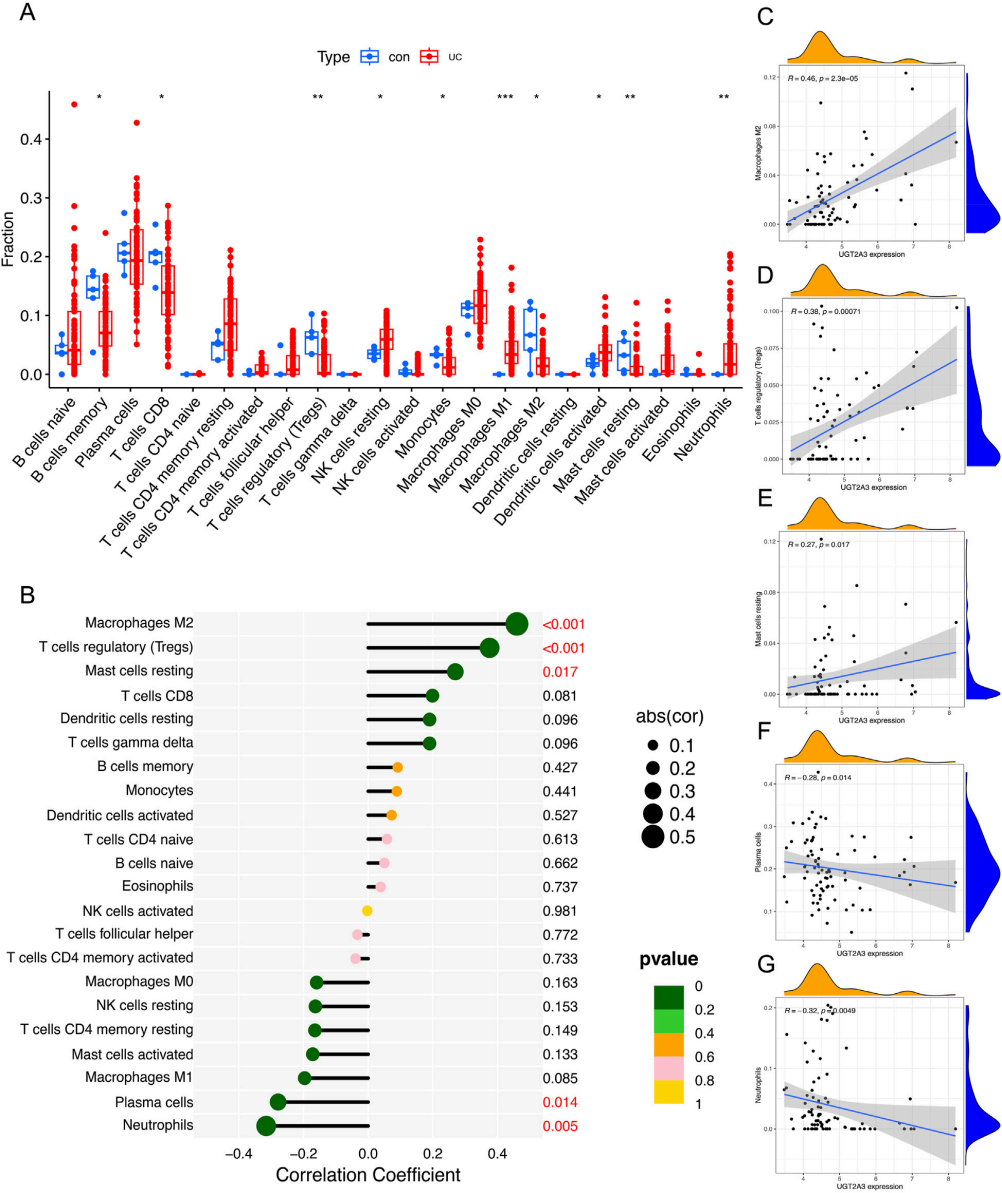
Immune infiltration. **(A)** Immune cell proportion in ulcerative colitis and normal tissues. **(B)** Correlation between *UGT2A3* expression and immune cells. Correlation of *UGT2A3* expression with Macrophages M2 **(C)**, T cells regulatory **(D)**, Mast cells resting **(E)**, Plasma cells **(F)**, and Neutrophils **(G)**. (*p < 0.05; **p < 0.01; ***p < 0.001).

Using the CIBERSORT method, the author calculated differences across 22 types of immune cells in the combined dataset, comparing ulcerative colitis samples with normal controls. The results showed significantly lower expression of memory B cells, CD8^+^ T cells, regulatory T cells (Tregs), monocytes, M2 macrophages, and resting mast cells in ulcerative colitis, whereas resting NK cells, M1 macrophages, activated dendritic cells, and neutrophils exhibited significantly higher expression (Figure 4A). Additionally, *UGT2A3* was positively correlated with M2 macrophages, Tregs, and resting mast cells, and negatively correlated with neutrophils and plasma cells (Figures 4B–G).

### 3.5 Validation in colorectal cancer

**FIGURE 5:**
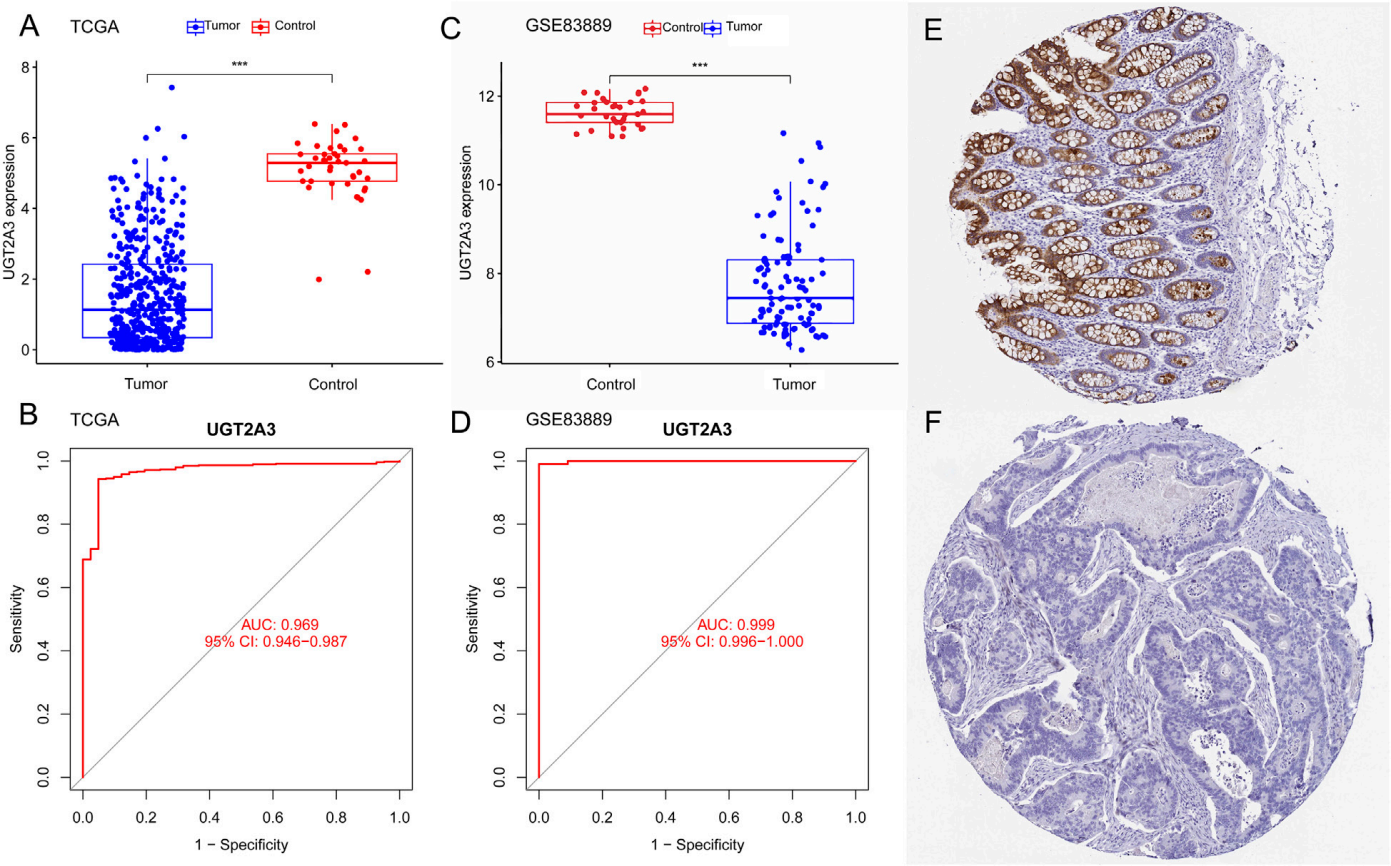
Expression differences and diagnostic value in colon cancer. **(A)** *UGT2A3* expression in colon cancer and normal tissues of TCGA dataset **(A)** and GSE83889 **(C)**. ROC curves of *UGT2A3* in TCGA dataset **(B)** and GSE83889 **(D)**. Protein expression of *UGT2A3* in normal tissue **(E)** and colon cancer tissue **(F)**. (*p < 0.05; **p < 0.01; ***p < 0.001).

The author used colorectal cancer gene expression data from the TCGA and GEO databases to validate *UGT2A3* expression differences between colorectal cancer and normal control groups. Results showed that *UGT2A3* was significantly downregulated in tumor tissues in both the TCGA COAD cohort (Figure 5A) and GSE83889 (Figure 5C), with excellent diagnostic effectiveness for colon cancer. Furthermore, *UGT2A3* protein was low expressed in colorectal cancer tissues in HPA data (Figures 5E, F). The AUCs for *UGT2A3* as a biomarker for colorectal cancer diagnosis in the TCGA dataset and GSE83889 were 0.969 (95% CI: 0.946–0.987) and 0.999 (95% CI: 0.996–1.000), respectively (Figures 5B, D).

### 3.6 Correlation between screened genes and clinical and immune factors

**FIGURE 6:**
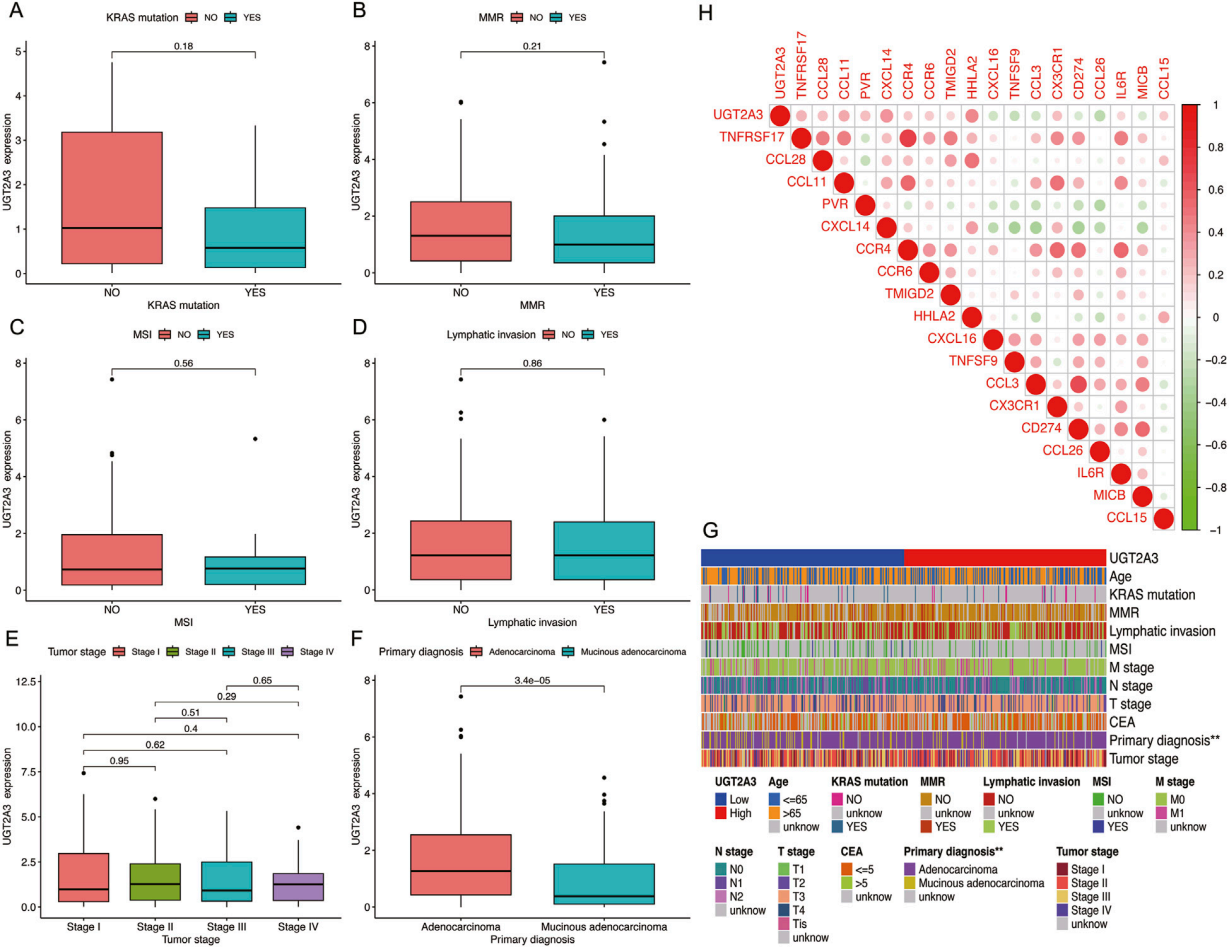
Correlation of *UGT2A3* expression with KRAS mutation **(A)**, MMR **(B)**, MSI **(C)**, Lymphatic invasion **(D)**, Tumor stage **(E)**, and primary diagnosis **(F)**. **(G)** Heatmap of *UGT2A3* expression and clinical indicator. **(H)** Relationships between *UGT2A3* expression and immune checkpoints.

The author analyzed the correlation between *UGT2A3* expression and clinical as well as immune indicators in colorectal cancer patients. Results indicate that *UGT2A3* is related to the primary diagnosis of colon cancer, with higher expression in adenocarcinoma and lower expression in mucinous adenocarcinoma (Figures 6A–G). Additionally, *UGT2A3* expression is correlated with CXCL14 and HHLA22 (Figure 6H).

### 3.7 Prognostic predictive power

**FIGURE 7:**
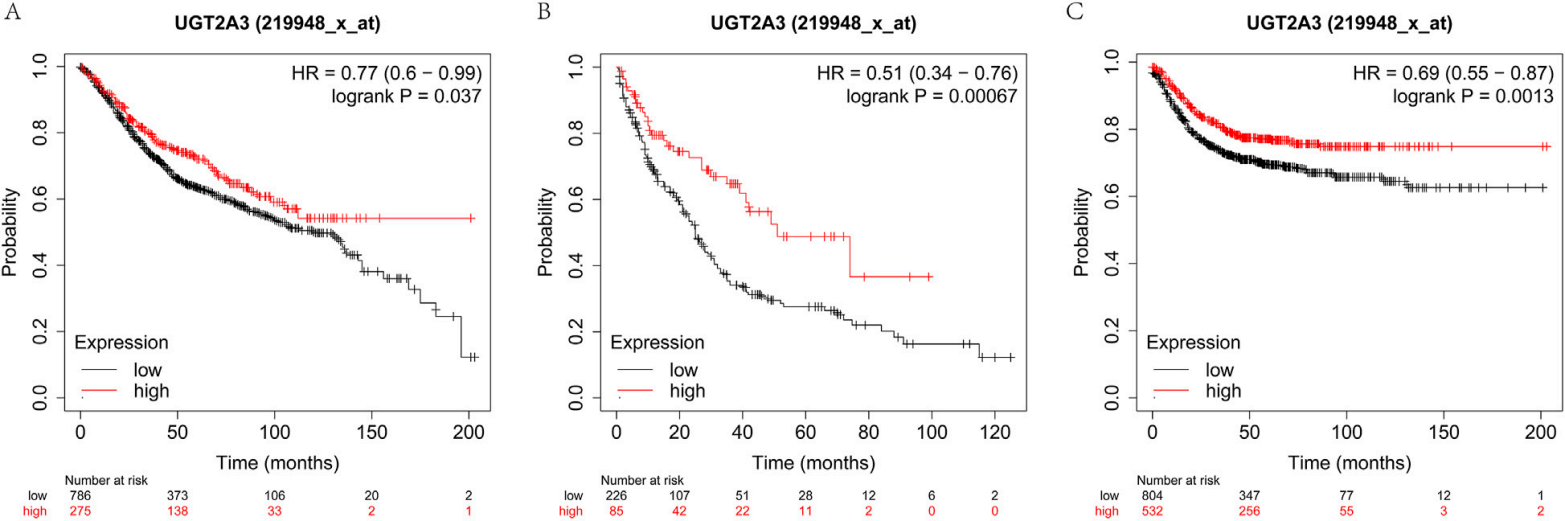
Survival difference according to *UGT2A3* expression. **(A)** Kaplan-Meier overall survival (OS) of colon cancer, **(B)** Kaplan-Meier post-progression survival (PPS) of colon cancer, **(C)** Kaplan-Meier recurrence-free survival of colon cancer.

The author assessed the impact of *UGT2A3* on the survival of colorectal cancer patients using the K-M database. Results indicate that *UGT2A3* expression is negatively correlated with OS, PPS, and RFS in colon cancer (Figures 7A–C).

## 4 Discussion

UDP-glucuronosyltransferase is an important metabolic enzyme in humans, capable of catalyzing the glucuronidation of numerous compounds into inactive polar derivatives for excretion in bile and urine (Mostaghel et al., 2016). Glucuronidation catalyzed by UGT is a crucial metabolic pathway, with almost all antitumor drugs being inactivated through this process. Research indicates that the glucuronidation of drugs can inactivate antitumor agents, potentially leading to drug resistance (Kiang et al., 2005; Malebari et al., 2017). Additionally, glucuronidation of certain oncogenic proteins can also lead to their inactivation (Raynal et al., 2010). Abnormalities or deficiencies of UGTs in the body are closely associated with certain diseases and drug reactions (Peters et al., 2003; Hu et al., 2016). UGTs also play a role in the development and progression of prostate cancer (du Toit and Swart, 2018; Park et al., 2006). And UGTs were reported to associate with sensitivity to various chemotherapy drugs, such as irinotecan, Etoposide and Tamoxifen (Meijerman et al., 2008). UGTs may also enhance patient tolerance to irinotecan through glucuronidation and potentially lead to better survival outcomes. Studies have also indicated that the UGT1A7 and/or UGT1A9 genotypes may be predictors of response and toxicity in colorectal cancer (CRC) patients treated with capecitabine and irinotecan (Carlini et al., 2005). UGTs are divided into four families: UGT1, UGT2, UGT3, and UGT8. *UGT2A3* is one of the proteins in the UGT family. Studies have confirmed that *UGT2A3* protein is stably expressed in the liver, intestines, and kidneys of humans (Gotoh-Saito et al., 2019). Studies indicate that *UGT2A3* could be associated with liver injury (Chen et al., 2020). It is still unclear how *UGT2A3* is related to ulcerative colitis.

Research indicates that the incidence of colorectal cancer in ulcerative colitis patients is 1.66 times greater than in the general population, and those with both CRC and UC face higher CRC mortality rates than patients with CRC alone (Olen et al., 2020; Lakatos and Lakatos, 2008). The study confirms that *UGT2A3* is low expressed in both ulcerative colitis and colorectal cancer, suggesting that *UGT2A3* may play a role in the development and progression of these disease. Some studies have demonstrated that upregulation of *UGT2A3* can inhibit the proliferation and metastasis of colorectal cancer cells, potentially mediated through miR-590-3p (Wu et al., 2023). *UGT2A3* has also been shown to be associated with the mortality rate in patients with COAD (Pang et al., 2019). The study shows that *UGT2A3* could be used as a diagnostic biomarker for colorectal cancer, exhibiting high diagnostic effectiveness. In the KM plot database, *UGT2A3* significantly influences survival, with patients who have high expression of *UGT2A3* experiencing better prognostic outcomes.

The author also found that *UGT2A3* is significantly positively correlated with M2 macrophages and Treg cells, and studies have shown that Macrophages M2 and Treg can promote tumor growth (Tanaka and Sakaguchi, 2017; Ruffell and Coussens, 2015). However, the significant expression of *UGT2A3* in COAD patients contradicts with our current understanding, suggesting that Macrophages M2 and Treg may not play a key role in the progression of UC to COAD. Further evidence is required to confirm this. Moreover, *UGT2A3* shows a significant negative correlation with neutrophils, with UC patients exhibiting extensive neutrophil infiltration (Pavlidis et al., 2022), low expression of *UGT2A3* suggests increased neutrophil infiltration, which could exacerbate inflammation in UC patients, thereby potentially promoting carcinogenesis (Zhang C. et al., 2023). Neutrophils contribute markedly to tissue damage and mucosal dysfunction in UC (Gupta et al., 2015). Research has shown that neutrophils are essential for tumor angiogenesis, and inhibiting tumor-associated neutrophils can significantly reduce vessel density (Bui et al., 2024). Additionally, researchers have reported that neutrophils can secrete IL-1 and promote colitis-associated tumorigenesis by activating the IL-1/IL-6 axis (Wang et al., 2014). Therefore, neutrophils play a critical role in the development of ulcerative colitis and colorectal tumorigenesis. The expression of *UGT2A3* is negatively correlated with neutrophil infiltration, suggesting that *UGT2A3* may influence ulcerative colitis and promote colitis-associated colorectal tumorigenesis by regulating neutrophil infiltration.

This study identified diagnostic markers for UC with excellent diagnostic effectiveness. The training set consists of two datasets which used same annotation platform, with a total of 106 patients. Although the sample size is small, it meets the criteria for performing LASSO regression and SVM-RFE, yielding convincing results. Consistent results were obtained in both ulcerative colitis and colorectal cancer samples, but the sample size of the ulcerative colitis validation datasets is small. Limitations included biases of data from public databases and lacking confirmatory experiments demonstrating the expressions and functions of *UGT2A3*.Additionally, the datasets have relatively few cases, and further validation with larger samples is needed. At the same time, the data used were obtained from public databases, with the limitations of restricted data types and fewer healthy controls.

## 5 Conclusion

The study shows that *UGT2A3* is significantly downregulated in both UC and CRC patients, making it a potential diagnostic marker for these conditions. As UC is a precancerous condition for CRC, *UGT2A3* may play a role in the progression of UC to CRC. The specific mechanism is not yet clear. The author intends to validate these results in cell lines and to investigate in animal models whether *UGT2A3* can drive the progression of colitis-associated colon cancer.

## Data Availability

The original contributions presented in the study are included in the article/supplementary material, further inquiries can be directed to the corresponding author.
